# Safety of BNT162b2 mRNA COVID‐19 Vaccine Batches: A Nationwide Cohort Study

**DOI:** 10.1002/pds.70207

**Published:** 2025-08-15

**Authors:** Anders Hviid, Ingrid Bech Svalgaard

**Affiliations:** ^1^ Department of Epidemiology Research Statens Serum Institut Copenhagen Denmark; ^2^ Pharmacovigilance Research Center, Department of Drug Design and Pharmacology, Faculty of Health and Medical Sciences University of Copenhagen Copenhagen Denmark

**Keywords:** batches, COVID‐19, vaccine safety

## Abstract

**Objectives:**

The safety of the BNT162b2 mRNA COVID‐19 vaccine has been extensively evaluated since the global rollout began. While serious adverse events are rare, safety issues continue to arise. This study evaluates the claim that earlier small vaccine batches were associated with higher rates of serious adverse events compared to later batches.

**Design, Participants, Setting:**

A nationwide cohort study was conducted in Denmark, comprising individuals vaccinated with the BNT162b2 vaccine from 52 pre‐defined batches classified into three pre‐defined groups. Vaccinated individuals were matched 1:1 between batch groups on age, sex, and vaccination priority group. The study outcomes included 27 serious adverse events, two negative control outcomes, and all‐cause mortality. Cox regression was used to estimate hazard ratios (HRs) comparing rates between batch groups in the 28 days following vaccination. We conducted two comparisons of the early small batches to two groups of larger batches used later in the pandemic.

**Results:**

In the study period, 9 983 728 vaccinations were administered from batches in the three pre‐defined groups. Slightly increased rates of arrhythmia were observed in both study comparisons, HRs 1.25 (95% CI, 1.05–1.50) and 1.15 (1.00–1.31), respectively, but sensitivity analyses did not robustly support these associations. For the remaining outcomes, increased rates in both study comparisons were not observed.

**Conclusion:**

This nationwide cohort study provides reassurance regarding the safety of the BNT162b2 vaccine across different batches used in Denmark. The findings support the overall safety of the vaccine, with no clinically relevant variations in serious adverse event rates between batches.

**Summary**

- Concerns about increased safety risks of early BNT162b2 vaccine batches originate from spontaneous adverse event reports.
- This large, nationwide Danish study found no robust evidence that early batches of the BNT162b2 vaccine were associated with higher rates of serious adverse events compared to later batches.
- Studies evaluating batch‐dependent safety with control groups are rare.
- A signal for arrhythmia was observed, but it was not consistent across comparison groups.
- The study supports the safety of the batches of BNT162b2 vaccine used during the study period in Denmark.

## 1 Introduction

The safety of the BNT162b2 mRNA COVID‐19 vaccine has been scrutinized extensively, and serious adverse events are rare. However, given the vast number of doses administered globally, it is to be expected that even rare serious adverse events occur shortly after vaccination purely by chance. Many pharmacovigilance reporting systems were initially overwhelmed by the large influx of reports of adverse events, including serious adverse events, at the start of the vaccination rollouts. This was a result of the sheer scale of the rollout, the call for all adverse events to be reported, and the fact that vulnerable elderly and healthcare workers were the first in line for vaccination.

A Danish group of researchers has questioned this interpretation of the initial spike in reports and has instead claimed that the earliest batches had safety issues which the later batches did not have [1]. This claim was based on data obtained through the Danish “public access to information”‐act. The data in question comprised the number of doses delivered according to batch numbers and the number of adverse events reported according to batch numbers. Their conclusion was that the smaller batches delivered early in the vaccination roll‐out had significantly higher rates of adverse events than larger batches delivered later in the roll‐out.

To evaluate the hypothesis that the earlier batches were associated with higher rates of serious adverse events and to circumvent the limitations of pharmacovigilance reports for causal assessments, we conducted a nationwide cohort study of the association between groups of batches and 30 outcomes: 27 diagnosed in the hospital setting, 2 negative control outcomes and all‐cause mortality.

## 2 Methods

### 2.1 Study Population

We designed a cohort study comprising all individuals living in Denmark and vaccinated at least once with a BNT162b2 vaccine (original monovalent version) from one of 52 pre‐defined batches. The 52 batches were the same ones as those included in a previous research letter based on data obtained through the Danish “public access to information”‐act [1, 2]. We verified that these batches matched batches recorded in the Danish Vaccination Register [3]. The batches were classified into three groups according to the those previously presented in the research letter: Group 1, with almost no adverse event reports (0–0.19 reports per 1000 doses), group 2 with slightly higher reporting rates (0.61–7.70 reports per 1000 doses), and group 3 with high reporting rates (14.7–184.6 reports per 1000 doses) [1]. We then matched the vaccinations in group 3 1:1 with group 1 and group 2, respectively. We used exact matching on age (10‐year intervals), sex and vaccination priority group (persons living in nursing homes, persons 65 years or older, who receive certain types of home care, selected patients with conditions that carry a significant increased risk of a severe course of COVID‐19, health care personnel and the general population). Age and sex was identified from the Danish Civil Registration System [4], while information on vaccination was obtained from the Danish Vaccination Register [3].

### 2.2 Outcomes

We included 30 study outcomes comprising 27 adverse events adapted from prioritized lists of adverse events of special interest for the COVID‐19 vaccines [5], two negative control outcomes and all‐cause mortality. Study outcomes were identified using International Classification of Disease 10th revision (ICD‐10) codes (Table S1) assigned discharge diagnoses for hospital contacts recorded in the Danish National Patient Register [6]. All‐cause mortality was identified from the Danish Civil Registration System [4]. We included primary diagnoses and inpatient, outpatient, and emergency department contacts. The date of admission served as the event date.

### 2.3 Statistical Analyses

The matched pairs were followed for 28 days for the occurrence of the study outcomes. Each of the 30 outcomes was studied separately, meaning that outcomes not under study did not censor follow‐up for the outcome under study. No individuals were eligible for matching if they had a history of the outcome in the 2 years preceding vaccination. Follow‐up was censored in the case of death, emigration, or disappearance from the national registers. We used Cox proportional hazards regression with time since vaccination as the underlying time scale to estimate hazard ratios (HRs) with 95% confidence intervals (CIs) comparing the hazards of the study outcomes in group 3 versus group 1 and group 2, respectively. Since individuals could contribute with multiple vaccinations, we used robust standard errors. We conducted sensitivity analyses in the form of age, sex, and priority group stratification of associations that were statistically significant in both comparisons.

## 3 Results

**FIGURE 1:**
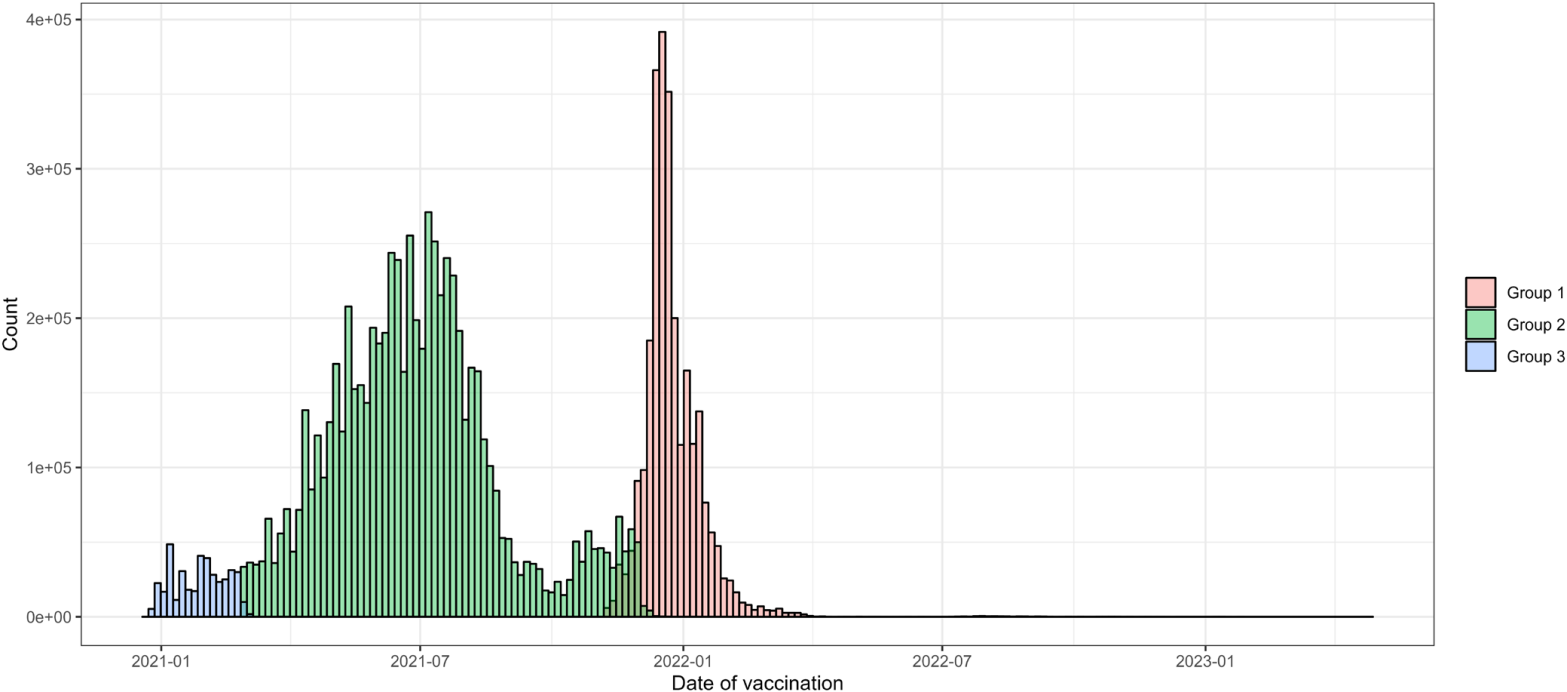
Distribution of vaccination dates for the three vaccine batch groups.

A total of 9 983 728 BNT162b2 vaccinations from the three batch groups were recorded; 2 647 879 (27%) from group 1, 6 935 006 (69%) from group 2, and 400 843 (4%) from group 3. The included vaccinations were administered from December 27, 2020, to April 25, 2023; although very few after March 2022 (Figure 1). Vaccinations from group 3 were administered from December 27, 2020, to October 28, 2022 (median date, January 30, 2021). Group 3 recipients were more likely to be elderly (33.4% were 80+‐year‐olds), female (68.2%) and had significant proportions of high‐risk individuals (10.1%) and frontline personnel (40.1%) (Table 1). Vaccinations from group 2 were administered from December 31, 2020, to November 11, 2022 (median date, June 26, 2021). Group 2 recipients were more likely to be middle‐aged individuals (34.7% were 40–59‐year‐olds) (Table 1). Vaccinations from group 1 were administered from January 7, 2021, to April 25, 2023 (median date, December 20, 2021). Group 1 recipients also had a high proportion of middle‐aged individuals (32.6% were 40–59‐year‐olds) (Table 1).

**TABLE 1:** Characteristics of each person in each vaccine batch group before matching and after matching (before outcome specific exclusions).

	Before matching			After matching	
	Group 1	Group 2	Group 3	Group 3 vs. 1	Group 3 vs. 2
	(*N* = 2 647 879)	(*N* = 6 935 006)	(*N* = 400 843)	(*N* = 479 570)	(*N* = 736 338)
Age					
0–9	156 705 (5.9%)	613 (0.0%)	4 (0.0%)	0 (0%)	0 (0%)
10–19	249 400 (9.4%)	794 564 (11.5%)	1579 (0.4%)	2956 (0.6%)	3138 (0.4%)
20–29	339 911 (12.8%)	827 855 (11.9%)	22 266 (5.6%)	44 032 (9.2%)	44 506 (6.0%)
30–39	217 601 (8.2%)	580 085 (8.4%)	33 615 (8.4%)	56 258 (11.7%)	67 208 (9.1%)
40–49	415 484 (15.7%)	1 054 970 (15.2%)	46 707 (11.7%)	68 724 (14.3%)	93 372 (12.7%)
50–59	502 421 (19.0%)	1 207 909 (17.4%)	57 447 (14.3%)	80 762 (16.8%)	114 408 (15.5%)
60–69	410 523 (15.5%)	1 033 284 (14.9%)	53 660 (13.4%)	66 260 (13.8%)	102 968 (14.0%)
70–79	291 559 (11.0%)	1 028 677 (14.8%)	51 528 (12.9%)	43 282 (9.0%)	89 258 (12.1%)
80–89	59 104 (2.2%)	366 154 (5.3%)	98 678 (24.6%)	106 956 (22.3%)	168 410 (22.9%)
90+	5171 (0.2%)	40 895 (0.6%)	35 359 (8.8%)	10 340 (2.2%)	53 070 (7.2%)
Sex					
Female	1 317 664 (49.8%)	3 490 389 (50.3%)	273 248 (68.2%)	326 304 (68.0%)	505 494 (68.6%)
Male	1 330 215 (50.2%)	3 444 617 (49.7%)	127 595 (31.8%)	153 266 (32.0%)	230 844 (31.4%)
Priority group					
Persons living in nursing homes	1287 (0.0%)	32 320 (0.5%)	61 867 (15.4%)	2558 (0.5%)	63 930 (8.7%)
Persons age ≥ 65, who receive certain types of home care	12 762 (0.5%)	53 816 (0.8%)	56 169 (14.0%)	25 500 (5.3%)	107 324 (14.6%)
Healthcare personnel	131 331 (5.0%)	401 563 (5.8%)	160 897 (40.1%)	248 886 (51.9%)	321 766 (43.7%)
Selected patients with significant increased risk of a severe course of COVID‐19	24 080 (0.9%)	173 564 (2.5%)	40 569 (10.1%)	48 010 (10.0%)	80 802 (11.0%)
Other	2 478 419 (93.6%)	6 273 743 (90.5%)	81 341 (20.3%)	154 616 (32.2%)	162 516 (22.1%)

We were able to match 239 785 pairs 1:1 in the group 3 versus 1 comparison (59.8% of group 3) and 368 169 pairs 1:1 in the group 3 versus 2 comparison (91.8% of group 3). The matched cohorts had higher proportions of elderly (~22% 80–89‐year‐olds), females (~68%), healthcare personnel (52% and 44% respectively), and high‐risk individuals (~10%) (Table 1).

**FIGURE 2:**
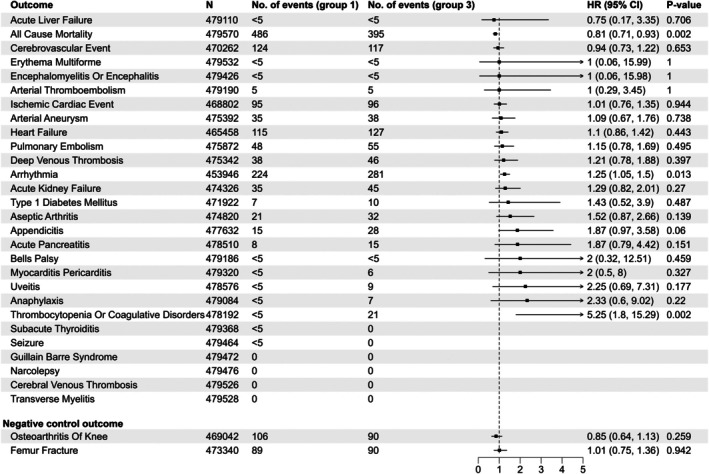
Main analysis comparing vaccine batch group 3 and vaccine batch group 1.

In the follow‐up for study outcomes in the group 3 versus group 1 comparison, we were able to include 453 832–479 528 individuals (depending on the specific study outcome and the number excluded due to a history of the outcome) (Figure 2) with a mean of 36 432.92 person‐years of follow‐up. The rates of cerebrovascular and ischemic cardiovascular events were not increased in group 3 compared to group 1, HRs 0.94 (95% CI, 0.73–1.22) and 1.01 (0.76–1.35), respectively (Figure 2). Out of the 28 study outcomes, only arrhythmia and thrombocytopenia and other coagulative disorders were observed at significantly higher rates in group 3, HRs 1.25 (95% CI, 1.05–1.50) and 5.25 (1.8–15.29). A number of study outcomes were very rare, and some, such as Guillain Barré syndrome and transverse myelitis, were not observed in either group 3 or group 1 (Figure 2). The HRs for both of the negative control outcomes were close to 1. All‐cause mortality was decreased, HR 0.81 (95% CI, 0.71–0.93).

**FIGURE 3:**
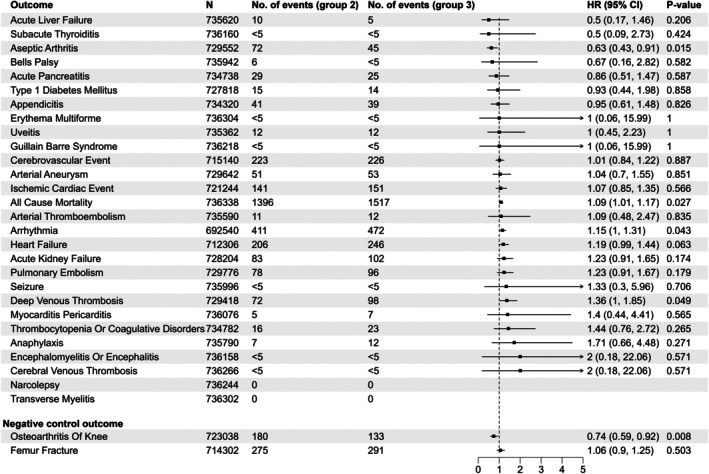
Main analysis comparing vaccine batch group 3 and vaccine batch group 2.

We were able to include 736 302–712 310 individuals in the group 3 versus 2 comparison (Figure 3) with a mean of 54 585.44 person‐years of follow‐up. The rates of cerebrovascular and ischemic cardiovascular events were not increased in group 3 compared to group 2, HRs 1.01 (95% CI, 0.84–1.22) and 1.07 (0.85–1.35), respectively (Figure 3). Out of the 28 study outcomes, only arrhythmia, deep vein thrombosis, and all‐cause mortality were observed at significantly higher rates in group 3 compared to group 2, HRs 1.15 (95% CI, 1.00–1.31), 1.36 (1.00–1.85), and 1.09 (1.01–1.17), respectively. The HR for the negative control outcome osteoarthritis of the knee was reduced in group 3 compared to group 2, HR 0.74 (95% CI, 0.59–0.92).

**FIGURE 4:**
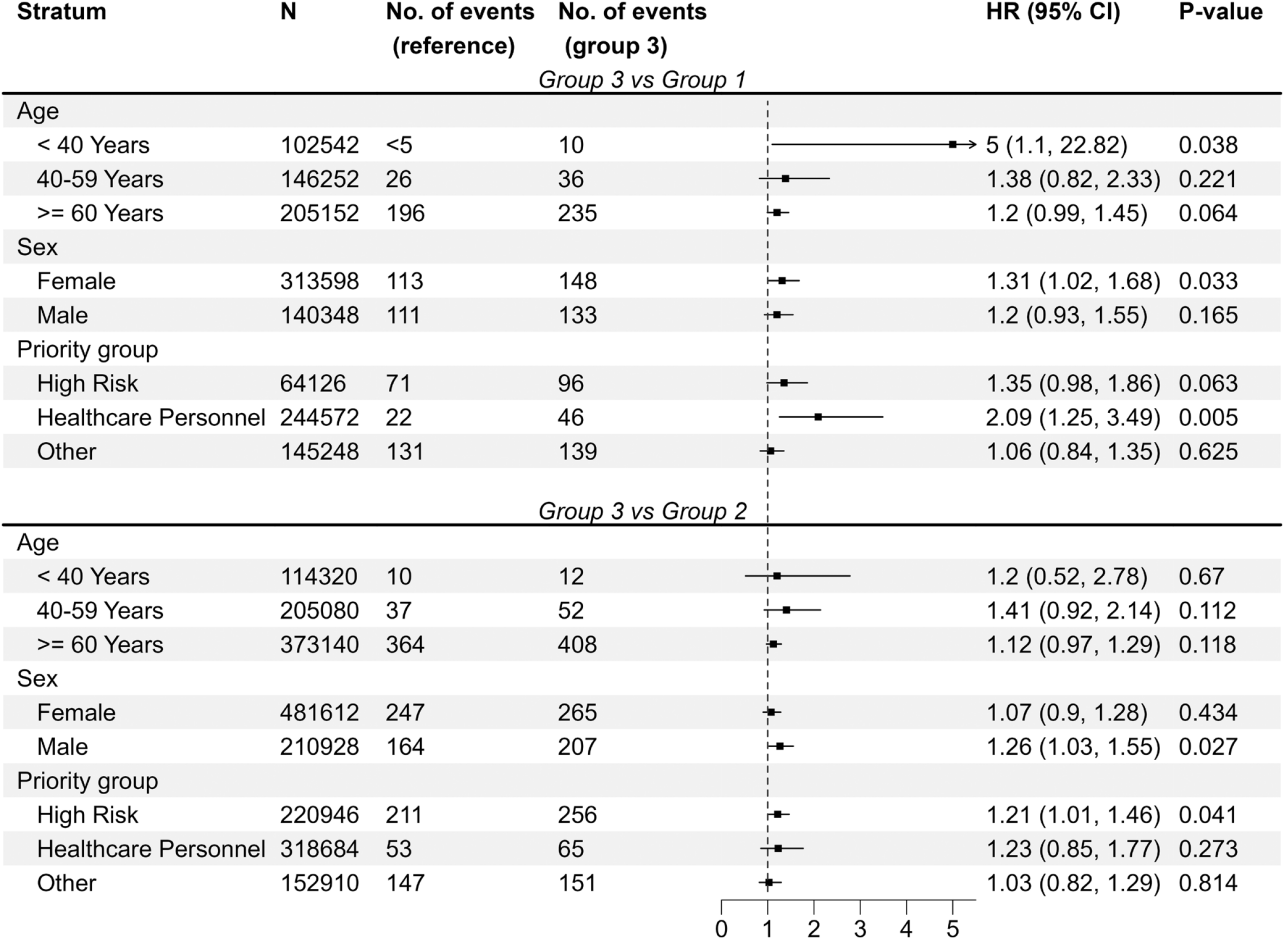
Stratification of hazard ratios of arrhythmia in both batch group comparisons.

We conducted post hoc sensitivity analyses of the association with arrhythmia in both comparisons (Figure 4). There was no consistent pattern between the two comparisons. In the group 3 versus group 1 comparison, the effect was largest among < 40‐year‐olds (HR 5.00, 95% CI, 1.10–22.82), females (1.31, 1.02–1.68), and health‐care personnel (2.09, 1.25–3.49), although confidence intervals were overlapping. In contrast, in the group 3 versus group 2 comparison, the effect was similar across age groups, between sexes, and between priority groups, with largely overlapping confidence intervals.

## 4 Discussion

Our findings suggest that the rates of serious adverse events, such as cerebrovascular and ischemic cardiovascular events, did not differ significantly across batch groups. The observations of slightly higher rates of arrhythmia in the first smaller batches of the vaccination roll‐out were not robust to sensitivity analyses.

The results align well with the current evidence emphasizing the overall safety of the COVID‐19 vaccines [5]; they are in contrast to the Danish research letter which claimed large variations in batch‐dependent safety [1]. The previous Danish study has a number of serious limitations [7, 8, 9]. The number of delivered vaccines does not equal administered vaccines within the study period. The study cut‐off date, 11 January 2022, means that a significant number of group 3 vaccinations would not even have been administered, let alone have resulted in adverse events and reports. The study also compares reporting rates in individuals and time periods that are not comparable. Group 3 comprised vulnerable elderly with multimorbidity and frontline personnel vaccinated at a time when the authorities recommended that all adverse events, including events such as sore shoulders and fever, were reported. Groups 1 and 2 comprised primary course‐ and booster vaccinations in the general population at a time when it was not recommended to report common and well‐known adverse events.

Our study circumvents many of the weaknesses of the previous research letter. We use diagnostic endpoints instead of pharmacovigilance reports and utilize a matched design which compares individuals of the same age, sex, and from the same vaccination priority group. However, our study also has a number of weaknesses. First, despite matching, residual confounding is still a possibility. Second, group 3 is more likely to be first doses, where group 2 comprises both first and second doses, and group 1 comprises booster doses. The reactogenicity of the vaccine may differ according to prior levels of immunity. Third and final, we are comparing vaccinations between different time periods. If there are strong calendar period trends in the study outcomes, we are not able to take this into account due to the almost perfect correlation between the batch groups and the periods in which they were administered. We believe this is reflected in the contrast of the all‐cause mortality results between comparisons. The slightly increased rate observed in the group 3 versus group 2 comparison is a comparison between vaccinations administered at a median date of January 30, 2021, and vaccinations administered at a median date of June 26, 2021, that is, a comparison between all‐cause mortality in winter and summer. In contrast, no increased rate was observed in the group 3 versus group 1 comparison, which is a comparison between vaccinations administered at a median date of January 30, 2021, and vaccinations administered at a median date of December 20, 2021, that is, a comparison of all‐cause mortality in two winter periods.

The batches included in this evaluation are not unique to Denmark, nor are the concerns about batch safety of the mRNA COVID‐19 vaccines. Variations between vaccine batches can theoretically arise from multiple factors throughout the lifecycle of a vaccine. These include the complex biological manufacturing processes, potential issues during storage and transportation, and aspects of clinical handling. This underscores the need for robust manufacturing controls and pharmacovigilance.

Our results from the first nationwide cohort study of batch safety with individual‐level data on vaccination and diagnoses provide reassurance that the safety of the BNT162b2 vaccine did not vary to any clinically relevant extent between batches used in Denmark between December 27, 2020 and April 25, 2023. Currently, there is no compelling evidence to suggest otherwise.

### 4.1 Plain Language Summary

Claims have been circulating that the first smaller batches of COVID‐19 vaccines might trigger more serious side effects than the larger batches that followed later in the pandemic. We conducted a Danish study to understand if people vaccinated with early batches of the Pfizer‐BioNTech COVID‐19 vaccine experienced more serious side effects than people vaccinated with later batches. We looked for 27 specific serious side effects in the medical records of people who received vaccines from different batches. Data on nearly 10 million vaccinations were included in our study. We concluded that early batches were not associated with more serious side effects than later batches. We did observe a slightly higher rate of heart rhythm problems associated with earlier batches, but this finding did not hold up in sensitivity analyses.

## Author Contributions

A.H. drafted the manuscript, supervised the study, and serves as a guarantor. I.B.S. carried out the statistical analyses, had full access to all the data in the study, and takes responsibility for the integrity of the data and the accuracy of the data analyses. All authors conceptualized the study, interpreted the results, and critically reviewed the manuscript. The corresponding author attests that all listed authors meet authorship criteria and that no others meeting the criteria have been omitted.

## Ethics Statement

The analyses were performed as surveillance activities as part of the advisory tasks of the governmental institution Statens Serum Institut (SSI) for the Danish Ministry of Health. SSI's purpose is to monitor and fight the spread of disease in accordance with section 222 of the Danish Health Act. According to Danish law, national surveillance activities conducted by SSI do not require approval from an ethics committee. Both the Danish governmental law firm and the compliance department of SSI have approved that the study is fully compliant with all legal, ethical, and IT‐security requirements; there are no further approval procedures required for such studies. No patients or members of the public were formally involved in defining the research question, study design, or outcome measures, or in the conduct of the study owing to privacy constraints, funding restrictions, and the short timeline during which the study was conducted.

## Conflicts of Interest

A.H. reports unrelated grants from Independent Research Fund Denmark, Lundbeck Foundation, and Novo Nordisk Foundation. A.H. is a Scientific Board Member of VAC4EU.

## Supporting information


**Data S1:** Supporting information.

## Data Availability

Owing to data privacy regulations in each country, the raw data cannot be shared.

## References

[1] M. Schmeling , V. Manniche , and P. R. Hansen , “Batch‐Dependent Safety of the BNT162b2 mRNA COVID‐19 Vaccine,” European Journal of Clinical Investigation 53 (2023): e13998. 37833825 10.1111/eci.14102

[2] V. Manniche , “Pfizer Corona Vacciner Kan Have Været Russisk Roulette–PRESSEMEDDELSE.” Vibeke Manniche Blog. https://web.archive.org/web/20231206140958/https://vibekemanniche.dk/pfizer‐corona‐vacciner‐kan‐have‐vaeret‐russisk‐roulette‐pressemeddelse/.

[3] T. Grove Krause , S. Jakobsen , M. Haarh , and K. Mølbak , “The Danish Vaccination Register,” Eurosurveillance 17 (2012): 20155. 22551494 10.2807/ese.17.17.20155-en

[4] C. B. Pedersen , “The Danish Civil Registration System,” Scandinavian Journal of Public Health 39 (2011): 22–25. 21775345 10.1177/1403494810387965

[5] N. W. Andersson , E. M. Thiesson , J. V. Hansen , and A. Hviid , “Safety of BA.4‐5 or BA.1 Bivalent mRNA Booster Vaccines: Nationwide Cohort Study,” BMJ 382 (2023): e075015. 37491031 10.1136/bmj-2023-075015 PMC10364193

[6] E. Lynge , J. L. Sandegaard , and M. Rebolj , “The Danish National Patient Register,” Scandinavian Journal of Public Health 39 (2011): 30–33. 21775347 10.1177/1403494811401482

[7] A. Hviid , “Inaccurate Representation of Shipped Vaccines as Administered,” European Journal of Clinical Investigation 53 (2023): e14066. 37452699 10.1111/eci.14066

[8] B. S. del Saz , “Batch‐Dependent Safety of the BNT162b2 mRNA COVID‐19 Vaccine,” European Journal of Clinical Investigation 53 (2023): e14050. 37833825 10.1111/eci.14102

[9] D. A. R. Scott , A. Renelle , and R. L. Niederer , “Methodological and Statistical Considerations: Batch‐Dependent Adverse Effects of COVID‐19 Vaccines,” European Journal of Clinical Investigation 53 (2023): e14073. 37548000 10.1111/eci.14073

