# Supplementary material for: Safety of BNT162b2 mRNA COVID‐19 Vaccine Batches: A Nationwide Cohort Study
Source: Pharmacoepidemiol Drug Saf. 2025 Aug 15;34(9):e70207. doi: 10.1002/pds.70207 (PMC12355449; doi:10.1002/pds.70207)
Supplement: Supplementary file 1 — Data S1: Supporting information. [file PDS-34-e70207-s001.docx]

**Supplementary material**

Safety of BNT162b2 mRNA COVID-19 vaccine batches: A nationwide cohort study

**Table of contents**

[Supplementary table S1: Outcome definitions 2](#_Toc158715935)

[Supplementary figure 1: Flowchart of study population 3](#_Toc158715936)

# **Supplementary table S1: Outcome definitions**

| **Outcomes** | **ICD-10 codes** |
| --- | --- |
| Anaphylaxis | T782, T783, T805, T886 |
| Ischemic cardiac event | I20-I251 |
| Cerebrovascular event | I60-66, G450-G453 |
| Arterial thromboembolism | I74 |
| Deep venous thrombosis | I80-82 (not I800, I808C, or I821) |
| Pulmonary embolism | I26 |
| Myocarditis or pericarditis | I300, I308, I309, I328, I401, I408, I409, I418, I514 |
| Cerebral venous thrombosis | I636, I676 |
| Thrombocytopenia or coagulative disorders | D65, D683, D686, D688-689, D690, D693-D699 (not D697 or D698A) |
| Guillain-Barré syndrome | G610 |
| Bell’s palsy | G510 |
| Transverse myelitis | G373 |
| Encephalomyelitis or encephalitis | G040, G040A, G048, G049, G058, G361 |
| Narcolepsy | G474 |
| Appendicitis | K35-K37 |
| Aseptic arthritis | M10, M119, M130, M131, M139 |
| Type 1 diabetes mellitus | E10 |
| Subacute thyroiditis | E061 |
| Heart failure | I110, I420, I426-I429, I50, J81 |
| Arrythmia | I44-I49 |
| Acute liver failure | K71, K72 |
| Acute kidney failure | D593, I12, I13, N00-N02, N04-N05, N08, N10, N141, N142, N144, N17, N19, R34 |
| Acute pancreatitis | K850, K853, K858, K859 |
| Erythema multiforme | L51 |
| Seizure | G40, G41 |
| Arterial Aneurysm | I71, I72 |
| Uveitis | H20, H30 |
|  |  |
| *Negative control outcomes* |  |
| Osteoarthritis of the knee | M17 |
| Femur fracture | S72 |

ICD-10 denotes International Classification of Diseases System, version 10. Only diagnoses registered as primary diagnoses were included in the outcome definitions.

# **Supplementary figure 1: Flowchart of study population**

**Not matched***
Group 3: n= 161,058
Group 1: n = 2,408,094

**Outcome-specific exclusion due to history of outcome**
Between n = 81 and n = 54,616

**Outcome-specific exclusion due to history of outcome**
Between n = 215 and n = 151,923

**Not matched***Group 3: n = 32,674
Group 2: n = 6,566,837

**Doses administered of a BNT162b2 vaccine from pre-defined batches**
N = 9,983,728

**Matched cohort (Group 3 and Group 2)***
N = 736,338

where

733,373 were followed for 28 days
2913 were censored due to death
52 were censored due to emigration
0 were censored due to disappearance

**Matched cohort (Group 3 and Group 1)***
N = 479,570

where

478,651 were followed for 28 days
881 were censored due to death
80 were censored due to emigration
0 were censored due to disappearance

* Indicates that numbers are *without* outcome-specific exclusion due to history of outcome. The number of included individuals for each outcome can be seen in Figure 2 and 3.
